# Evaluation of BAYESIL for automated annotation of ^1^H NMR data using limited sample volumes: application to African elephant serum

**DOI:** 10.1007/s11306-023-02001-1

**Published:** 2023-03-30

**Authors:** Christiaan De Wet van Zyl, Mari van Reenen, Gernot Osthoff, Ilse du Preez

**Affiliations:** 1 Centre for Human Metabolomics, North-West University, Potchefstroom, South Africa; 2 Department of Microbiology and Biochemistry, University of the Free State, Bloemfontein, South Africa

**Keywords:** BAYESIL, ^1^H NMR, Limited sample volume, Serum, Elephant

## Abstract

**Introduction:**

Technological advancements enabled the analyses of limited sample volumes on ^1^H NMR. Manual spectral profiling of the data is, however, complex, and timely.

**Objective:**

To evaluate the performance of BAYESIL for automated identification and quantification of ^1^H NMR spectra of limited volume samples.

**Method:**

Aliquots of a pooled African elephant serum sample were analyzed using standard and reduced volumes. Performance was evaluated on confidence scores, non-detects and laboratory CV.

**Results:**

Of the 47 compounds detected, 28 had favorable performances. The approach could differentiate samples based on biological variation.

**Conclusions:**

BAYESIL is valuable for limited sample ^1^H NMR data analyses.

**Supplementary Information:**

The online version contains supplementary material available at 10.1007/s11306-023-02001-1.

## Introduction

Metabolomics is defined as the measurement of the changes in the low molecular weight compounds (metabolites) in a specific biological specimen because of internal fluctuations and/or external disturbances (Lenz & Wilson, 2007). Metabolomics is well established in animal studies including species such as fish (Tavares et al., 2022), mussels (Boaz et al., 2012; Wu & Wang, 2010), mice (Mason et al., 2018), rats (Maulidiani et al., 2017) seals (Boaz et al., 2012) and more. For this reason, researchers are striving to optimize and standardize protocols for the analyses of biological specimens collected from specific animal species, such as was recently done for the proton nuclear magnetic resonance (^1^H NMR) analyses of whole blood (Lenz & Wilson, 2007; Wood et al., 2022) and milk (Osthoff et al., 2023) collected from African elephants. The use of ^1^H NMR as an analytical tool for metabolomics analysis has many advantages, including the fact that it requires minimal, low cost sample preparation, and is robust, rapid, unbiased, reproducible, non-selective, non-destructive and quantitative (Lenz & Wilson, 2007). This technique, nevertheless, has a low sensitivity when compared to other methods, such as mass spectroscopy, and therefore typically requires larger sample volumes. Advancements in the hardware technology, and the development of systems such as the MATCH adapter, have, however, facilitated the optimization of limited sample volume analyses (Mason et al., 2018). A drawback of ^1^H NMR in untargeted metabolomics studies, is the high complexity of spectral profiling, specifically the annotation and quantification of metabolites from spectra. The processing is time consuming, labor intensive, requires expert skills and is subjective and prone to human error (Tredwell et al., 2011). BAYESIL was developed as an automated alternative to manual ^1^H NMR spectral profiling (Ravanbakhsh et al., 2015). The freeware automatically quantifies metabolites, which are identified with specified confidence based on a match to a built-in library, in less than 5 min per spectrum (Ravanbakhsh et al., 2015). This open source software has been evaluated and deemed useful for metabolomics applications by various groups. Such assessments typically entail comparisons between BAYESIL and manual spectral profiling (Tavares et al., 2022), or between BAYESIL and other automated software tools (Lipfert et al., 2019; Maulidiani et al., 2017). In these studies, the prescribed sample volumes and preparation methods are used. For serum this includes filtering, buffering and the addition of reference standards. It is suggested that a total serum volume, after filtration, of 540 µL is used per sample. The aim of our study was to evaluate the performance of BAYESIL when using a reduced sample volume of 54 µL, after filtration (10% of that stipulated in the protocol). The ability of the miniaturized approach to differentiate between sample groups was subsequently explored by graphically presenting metabolite differences between serum collected from female (lactating and using contraceptives) and male African elephants and by evaluating their biological relevance. Outputs from the study would give an indication of the sensitivity that can be expected when spectra are processed by a non-expert, and would stipulate the feasibility of automated, high-throughput, limited sample volume, ^1^H NMR analyses. To the best of our knowledge, this is the first evaluation of the freeware using very low sample volumes. In addition, although BAYESIL has been used as a tool in various studies using both human (Garg et al., 2018; Grimaldi et al., 2018; Maulidiani et al., 2017) and animal samples (Tavares et al., 2022), this study is the first to assess its value in African elephant research.

## Materials and methods

### Animals and sample collection

Blood samples were obtained from two female lactating African elephants (*Loxodonta africana*) (1) Mussina at 15, 16, 16.3, 16.5, 17.3 and 17.7, and (2) Shan at 27.5, 27.9, 28.1, 29.1 and 29.4 months of lactation. In addition, blood was also collected at six time points, roughly 2 weeks apart, from the same two elephants (after lactation), from a third female, Naledi, and two males, Chova and Chishuru. The three non-lactating female animals were on hormonal contraceptives at the time of sample collection. The elephants roamed free in the Adventures with Elephants Reserve (Bela Bela, Limpopo province, South Africa). The nutrition, care and well-being of the animals were described in a previous paper (Kobeni et al., 2020). Each blood sample was drawn from a posterior auricular vein through a 12-gauge needle into an 8.5ml yellow top vacuum tube. The tubes were kept on ice for 30 min during transportation to the laboratory, and the serum was frozen at -80 ℃ until further analysis.

### Quality control samples

Metabolomics analyses were conducted at the Centre for Human Metabolomics, North-West University, South Africa. To assess the miniaturized method’s performance, in comparison to the standard BAYESIL method, a pooled quality control (QC) sample was compiled by combining 200 µL of each animal’s serum sample. Aliquots of this QC sample were analyzed over three days (three aliquots per day, each prepared separately), using both sample preparation methods. To determine the sample stability of each method, one prepared QC aliquot of both methods was consecutively analyzed on the ^1^H NMR, once every hour, for 24 h.

### ^1^H NMR buffer preparation

The ^1^H NMR buffer was prepared according to BAYESIL instructions, with the negligeable modification of using potassium formate instead of the prescribed sodium formate. Also, sodium azide was added to prevent possible bacterial growth. In short, to prepare 100 mL buffer, 20.4 g monobasic potassium phosphate (final concentration in sample is 150 nM); 100 mg trimethylsilylpropanoic acid (TSP, 580.5 μm); 8.4 mg potassium formate (100 nM) and 2 mL of a 100 mM sodium azide solution was added to a volumetric flask, which was then filled to 100 mL with deuterated water (D_2_O). The final pH was adjusted to 7 with potassium hydroxide.

### Standard sample preparation

For the standard sample preparation, the method suggested by BAYESIL was applied. Instead of the 3 500 molecular weight cutoff MWCO centrifugal units recommended by BAYESIL, 10 000 molecular weight cutoff (MWCO) centrifugal units, which are readily available in our laboratory, were used. Of each QC aliquot, 800 µL was added to an Amicon Ultra-2mL 10 000 MWCO centrifugal unit (5 x pre-rinsed with water) and centrifuged at 2860 x g for 40 min. From the filtrate, a volume of 540 µL was added to a microcentrifuge tube that contained 60 µL of the ^1^H NMR buffer solution (9:1 ratio). After a brief vortex step, 600 µL of the buffered filtrate was transferred to a 5 mm ^1^H NMR tube and sealed with a cap.

### Miniaturized sample preparation

For the preparation of samples in reduced volumes, a previously described method (Mason et al., 2018) was slightly modified. Briefly, 100 µL of each QC aliquot and analytical sample was added to a Centrifree-1mL 10 000 MWCO centrifugal filter (5 x pre-rinsed with water) which was centrifuged at 2860 x g for 40 min. The filtrate (54 µL) and 6 µL ^1^H NMR buffer (9:1 ratio) was added to a 2 mm ^1^H NMR tube and mixed, using a preprogrammed eVol® NMR digital syringe with a 180-mm-long bevel-tipped needle. An adapter with a gripper (Bruker MATCH system) was used to hold the 2 mm NMR tube to be analyzed in a 5 mm NMR probe.

### Data acquisition

Prepared QC aliquots and samples were loaded onto a SampleXpress autosampler. After QC analyses for method performance testing, animal samples were analyzed in a single batch in randomized order. Samples were measured at 500 MHz on a Bruker Avance III HD NMR spectrometer equipped with a 5 mm triple-resonance inverse (TXI) ^1^H {15 N, 13 C} probe head and x, y, z gradient coils. The inner coil of the TXI probe was optimized for ^1^H observation. ^1^H spectra were acquired as 128 transients in 32 K data points with a spectral width of 10,504 Hz for the standard method and 6000 Hz for the miniaturized method, with an acquisition time of 3.2s for the standard method and 2.72s for the miniaturized method. Receiver gain was set at 90.5. The sample temperature was maintained at 300 K, and the H_2_O resonance was pre-saturated by single-frequency irradiation during a relaxation delay of 4 s, with a 90° excitation pulse of 8 µs. Shimming of the sample was performed automatically on the deuterium signal. The quality of the spectra was checked by ensuring that resonance line widths for TSP and the metabolites were < 1 Hz. Software used for NMR pre-processing was Bruker Topspin (V3.6.4).

### Data processing

After acquisition of spectra, each QC aliquot and sample spectral data file was converted and uploaded to BAYESIL according to the instructions. The following options on BAYESIL were selected: serum biofluid profile; TSP as chemical shift reference with a concentration of 580.5 µM; 500 MHz NMR frequency, and standard speed analysis. The output data from BAYESIL for each QC aliquot and sample were transferred to a single excel sheet.

### Statistical analysis

To quantify performance, the data obtained from BAYESIL for the nine QC aliquot analyses (three batches with three analyses each) for each sample preparation method, were analyzed using Microsoft Excel and SPSS. The performance of both methods was evaluated for each metabolite based on the identification confidence score (CS) assigned by BAYESIL; the number of zero concentration values (non-detects); the precision (laboratory coefficient of variation (CV)); stability; and traceability. Metabolites were only retained and evaluated across the elephant groups if they had a CS > 7 across all spectra; no non-detects, and a laboratory CV < 20% for the miniaturized method (Kaza et al., 2019). The laboratory CV was calculated as the laboratory standard deviation, which is the variance components or the weighted sum of the between and within batch variability, expressed as a percentage of the average. Sample stability was evaluated by calculating the Pearson correlation coefficient between observations and run order but was not used as a measure to exclude metabolites from further analyses. To determine a potential loss of traceability, the concentrations of the retained metabolites as measured with the miniatured method, was plotted against the corresponding concentration measured with the standard method, and the coefficient of determination (R^2^) was calculated. To evaluate the miniaturized method’s ability to identify variations between biological sample groups, box plots of the measured concentrations of the retained metabolites were constructed using R (version 4.2.0). Given the small group sizes, no further statistical analyses were performed.

## Results and discussion

### Method performance

A summary of the performance of both sample preparation methods is given in Table 1 (details are provided in supplementary material Table S1). All 47 metabolites in the BAYESIL serum reference library were identified in all samples using both methods. The CS values, indicating a degree of certainty of the compound annotation, ranging between 0 and 10, with 10 being the surest, was similar across the individual compounds for both methods. Although most metabolites had favorable CSs, 13% (six metabolites) had a CS < 7 and were excluded from further analyses, retaining 41 metabolites. When considering the percentage of non-detects, the performance of the methods was comparable. The overall percentage of non-detects was 5% and 7% for the miniaturized and standard methods respectively. The miniaturized method therefore had a larger number of compounds without any non-detects. Only metabolites without any non-detects across all QC aliquots were considered for evaluating precision, retaining 37 for the miniaturized method and 35 for the standard method. Precision distributions of the methods were comparable, ranging between 6 and 65% and 12–74% for the miniaturized and standard methods respectively. After further removal of metabolites with undesirable precision (laboratory CV > 20%), 28 and 20 metabolites were retained for the miniaturized and standard methods, respectively. The 24-hour stability experiments only indicated random variability and not trends in time (Pearson’s | r | <0.8) with the standard method proving superior with CVs below 20%. Finally, the observed concentrations, as detected with both methods, of the retained metabolites (based on the miniaturized method performance) were compared to identify any loss of traceability (supplementary material Figure S1). The concentrations generated by the miniaturized method compared extremely well to those produced by the standard method, with an R^2^ of 1 when considering all metabolites retained, and an R^2^ of 0.992 when considering only the metabolites in the lower concentration range. The miniaturized method showed a slight loss of sensitivity with the intercepts of -0.8 (overall) and − 1.98 (lower concentrations).

**Table 1:** Summary of method performance

Metabolite Name	CS	Miniaturized Method		Standard Method		Metabolite Name	CS	Miniaturized Method		Standard Method	
		ND	Lab CV	ND	Lab CV			ND	Lab CV	ND	Lab CV
L-Asparagine	X	✔	X	✔	X	**Acetic acid**	✔	✔	✔	✔	✔
L-Serine	X	✔	✔	✔	X	**Isobutyric acid**	✔	✔	✔	✔	X
1-Methylhistidine	X	✔	X	✔	X	**Formic acid**	✔	✔	✔	✔	✔
L-Tryptophan	X	✔	X	✔	X	**Methanol**	✔	✔	✔	✔	✔
L-Arginine	X	✔	X	X	X	**Ethanol**	✔	✔	✔	✔	X
Ornithine	X	X	X	X	X	**2-Hydroxybutyrate**	✔	✔	✔	✔	✔
Choline	✔	X	X	X	X	**D-Glucose**	✔	✔	✔	✔	✔
Propylene glycol	✔	X	X	X	X	**Betaine**	✔	✔	✔	✔	✔
Isopropanol	✔	X	X	X	X	**Succinic acid**	✔	✔	✔	✔	✔
Glycerol	✔	X	X	✔	X	**L-Alanine**	✔	✔	✔	✔	✔
L-Proline	✔	✔	X	✔	X	**L-Glutamic acid**	✔	✔	✔	✔	X
Urea	✔	✔	X	✔	X	**Creatinine**	✔	✔	✔	✔	✔
Malonic acid	✔	✔	X	X	X	**Hypoxanthine**	✔	✔	✔	✔	✔
3-Hydroxybutyric acid	✔	✔	X	X	X	**L-Valine**	✔	✔	✔	✔	✔
L-Threonine	✔	✔	X	✔	X	**L-Tyrosine**	✔	✔	✔	✔	✔
L-Methionine	✔	✔	X	✔	X	**L-Leucine**	✔	✔	✔	✔	✔
L-Carnitine	✔	✔	X	X	X	**Creatine**	✔	✔	✔	✔	✔
L-Histidine	✔	✔	X	✔	X	**Lysine**	✔	✔	✔	✔	X
L-Phenylalanine	✔	✔	X	✔	✔	**Acetoacetate**	✔	✔	✔	✔	✔
**Glycine**	✔	✔	✔	✔	✔	**Citric acid**	✔	✔	✔	✔	✔
**L-Isoleucine**	✔	✔	✔	✔	X	**L-Glutamine**	✔	✔	✔	✔	✔
**Acetone**	✔	✔	✔	✔	X	**Dimethylsulfone**	✔	✔	✔	✔	X
**L-Aspartic acid**	✔	✔	✔	✔	X	**L-Lactic acid**	✔	✔	✔	✔	✔
**Pyruvic acid**	✔	✔	✔	✔	X						

✔ Indicates a favourable measurement (CS > 7; no NDs; lab CV < 20%). X indicates an unfavourable measurement.

Compounds in bold were retained and evaluated in animal sample data.

CS: confidence score; ND: non-detects.

### Biological application

**Fig. 1:**
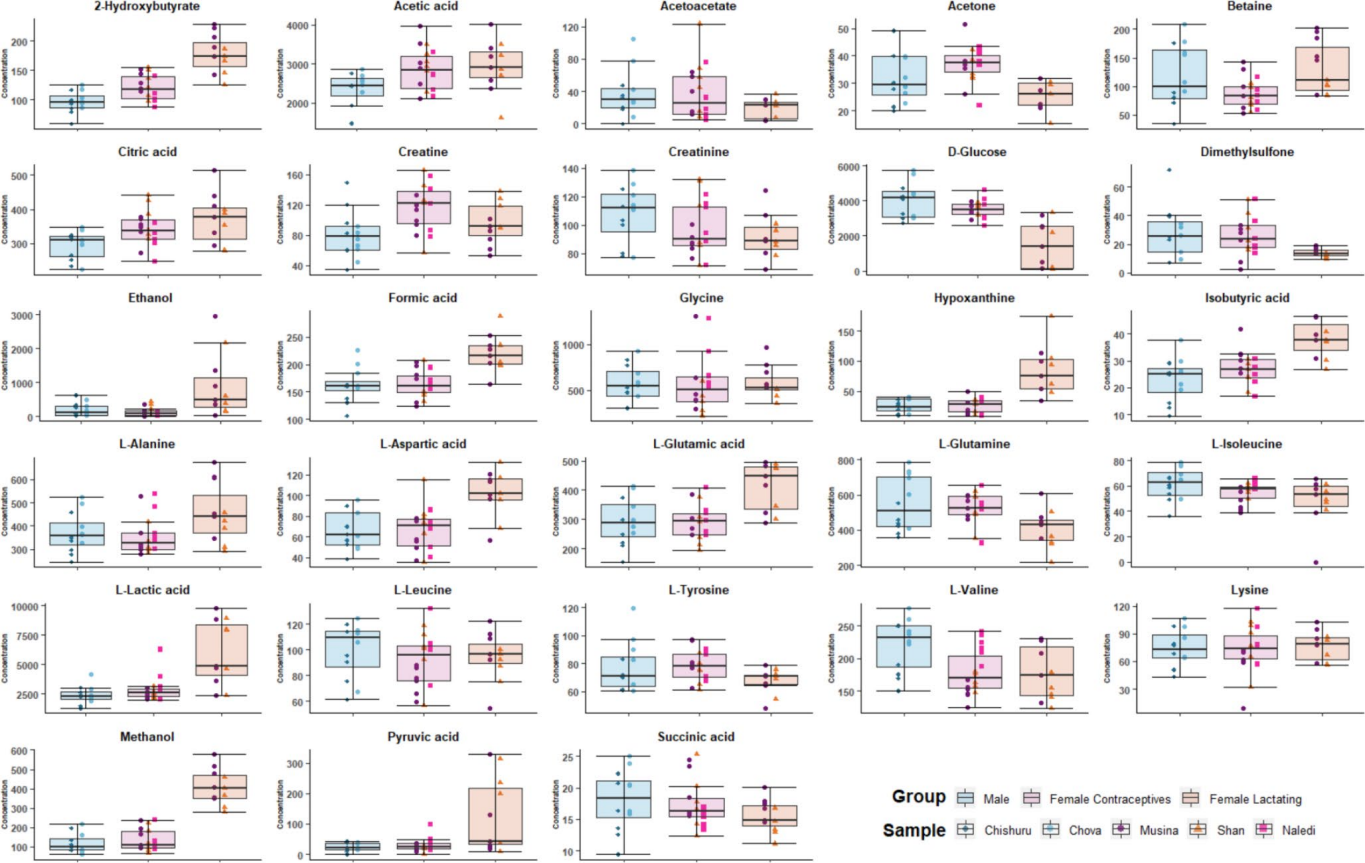
Boxplots of the metabolites retained based on the performance outcome of the miniaturized method

Metabolites were only retained for investigation in the animal samples based on the performance outcome of the miniaturized method. Figure 1 shows the box plots of the retained metabolites, indicating the distribution of the data in each sample group. Since metabolomics investigations in African elephants are scarce, reference to the human and other animal mechanisms will also be used where appropriate to substantiate the metabolome variations detected between the sample groups.

Several sex-related variations in the serum metabolic profile of healthy humans have been identified. One study, for example, detected comparatively higher concentrations in the complete spectrum of amino acids and carbohydrates in males (Krumsiek et al., 2015). Here, the mean concentrations of the branched chain amino acids specifically (isoleucine, leucine, and valine), as well as creatine and creatinine, were slightly higher in the male elephant group, compared to the female groups, which could perhaps be ascribed to a higher proportion of muscle mass. However, in a recent study, no differences attributed to sex were found in the whole blood metabolomes of healthy African elephants (Wood et al., 2022). The non-lactating female group used in our study was on hormonal contraceptives at the time of sampling, which could be seen as a potential confounding factor when comparing the sexes. When investigating the human serum amino acid profile, it was found that contraceptive use induced: (1) a decrease in alanine, glutamine, glycine, isoleucine, tyrosine and valine; (2) an increase in glutamic acid and aspartic acid and 3) no significant variation in lysine (Swanepoel et al., 2021). Except for glutamine and tyrosine, the same profile, indicating an overall increase in the oxidative stress levels, was detected in our female contraceptive group, compared to the males. As would be expected, differences were also observed between the two female groups. It is well known that lactation induces major maternal metabolic variations as a means to meet the greater need for lipids and glucose for milk production (Gunderson, 2014), and up to 50% higher rates of glycogenolysis have been reported to meet the glucose demand in breast feeding women (Tigas et al., 2002). Accordingly, variations in the levels of various saccharides, and an increased concentration of glutamate, in the form of a glucogenic amino acid, indicating an upregulated saccharide synthesis, were detected in the milk of African elephants over the course of lactation (Osthoff et al., 2023). Here, we detected lower glucose concentrations in the serum of the lactating animals, compared to the male and female contraceptive groups. A study conducted in Sprague-Dawley rats further indicated an increase in the expression of glycolysis enzymes with a simultaneous down regulation in the Krebs cycle and significant induction of lactate dehydrogenase as a means to increase lactate (Xiao et al., 2004). This coincides with the variations detected in the concentrations of pyruvic acid, citric acid, succinic acid, and lactic acid in the lactating female elephants compared to the other groups. Furthermore, 2-hydroxybutyrate (Ferrannini et al., 2013), which is an indicator of insulin resistance, was detected in comparatively higher concentrations in the lactating animals.

These results therefore indicate that differences of biological importance can be detected with this automated profiling tool, using limited serum sample volume. The outcome also supports future metabolomics studies investigating metabolic variations in African elephants to better elucidate mechanisms of reproduction and lactation.

## Conclusions

The ability to reliably analyze limited samples volumes in metabolomics studies is crucial, and would facilitate the analyses of scares samples, such as in the case of terminally ill patients, or samples which are difficult to collect, such as from free roaming elephants. Automated data analysis further promotes results with higher accuracy and precision, which can rapidly be acquired, even by a non-expert. We do, however, recommend that the application of this approach is verified prior to each metabolomics study, to evaluate the performance of the method for the specific sample matrix and laboratory.

## Electronic supplementary material

Below is the link to the electronic supplementary material.

Supplementary Material 1

## Data Availability

Raw data were generated at the Centre for Human Metabolomics, North-West University, South Africa. Derived data supporting the findings of this study are available at https://www.ebi.ac.uk/biostudies/studies/S-BSST1045 (accession number: S-BSST1045).
