# Supplementary material for: Evaluation of BAYESIL for automated annotation of 1H NMR data using limited sample volumes: application to African elephant serum
Source: Metabolomics. 2023 Mar 30;19(4):31. doi: 10.1007/s11306-023-02001-1 (PMC10063514; doi:10.1007/s11306-023-02001-1)

Supplementary information to:

**Evaluation of BAYESIL for automated annotation of ^1^H NMR data using limited sample volumes: Application to African elephant serum**

Christiaan De Wet van Zyl^1^, Mari van Reenen^1^, Gernot Osthoff^2^, Ilse du Preez^1*^

^1^Centre for Human Metabolomics, North-West University, Potchefstroom, South Africa;

^2^Department of Microbiology and Biochemistry, University of the Free State, Bloemfontein, South Africa


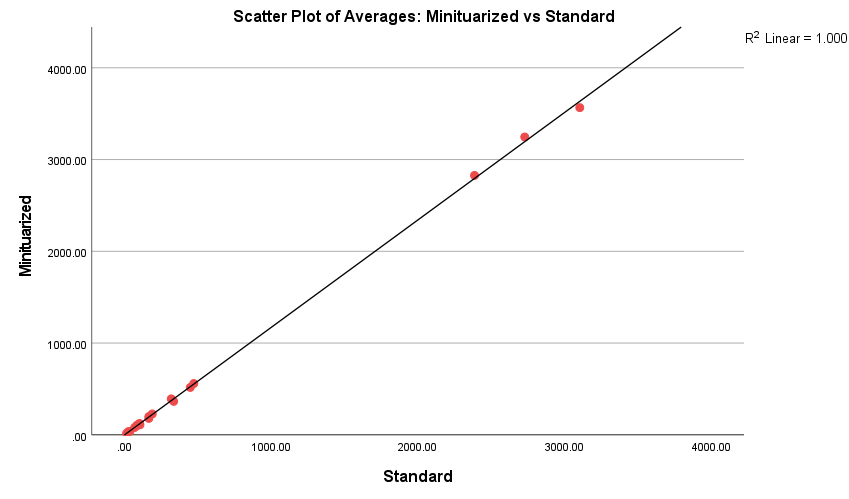

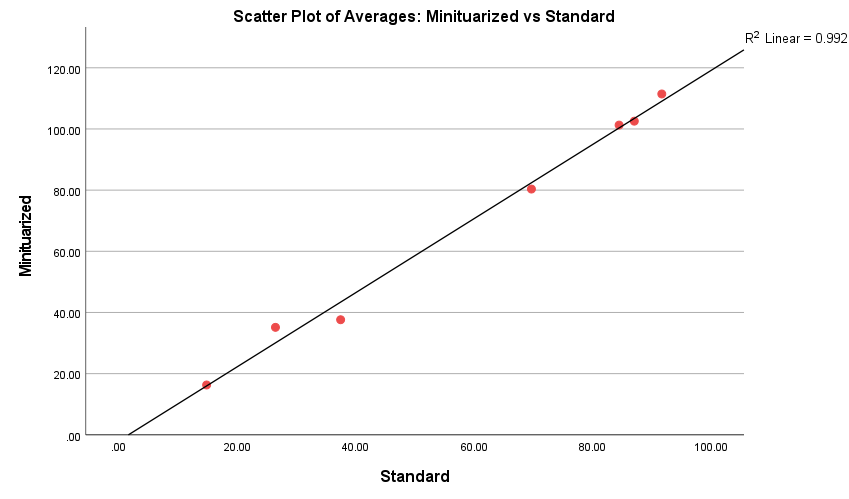


*Figure S1: Comparison of the concentrations of the metabolites measured after application of both sample preparation methods. Left: all metabolites retained based on the miniaturized method performance. Right: Metabolites in the lower concentration range.*

Table S1: Details of method performance


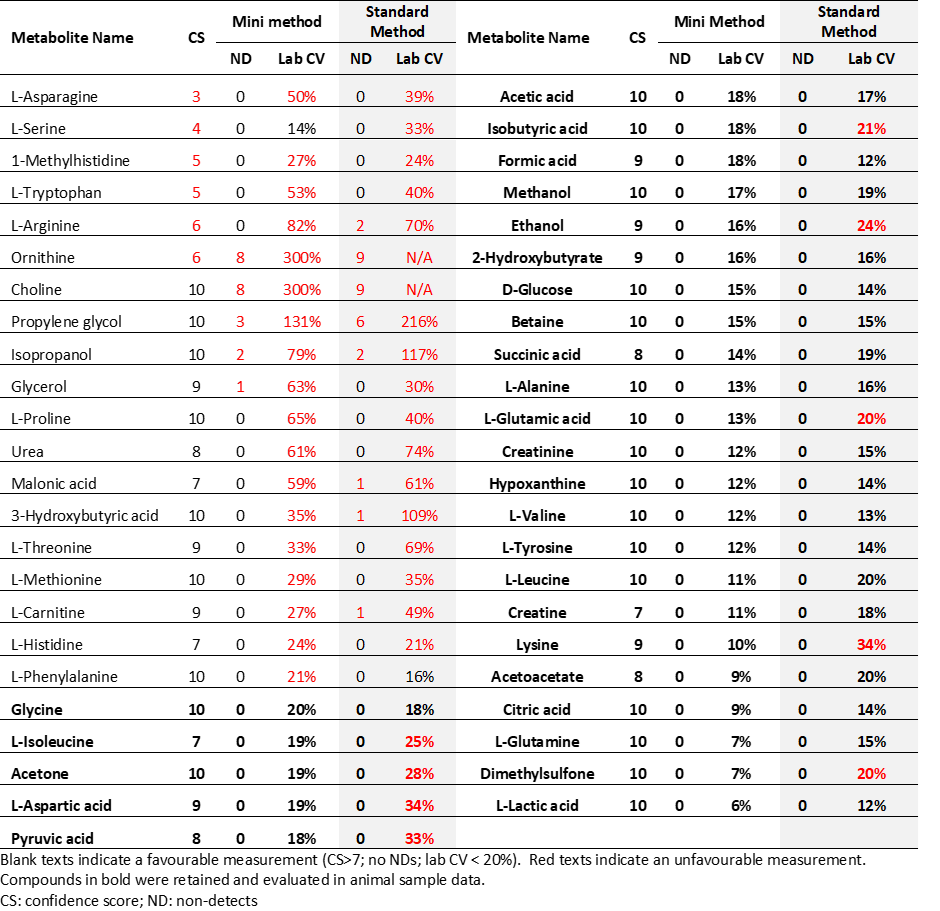

Supplement: Supplementary file 1 — Supplementary Material 1 [file 11306_2023_2001_MOESM1_ESM.docx]
